# Supplementary material for: Parvalbumin basket cell myelination accumulates axonal mitochondria to internodes
Source: Nat Commun. 2022 Dec 9;13:7598. doi: 10.1038/s41467-022-35350-x (PMC9734141; doi:10.1038/s41467-022-35350-x)
Supplement: Supplementary file 1 — Supplementary Information [file 41467_2022_35350_MOESM1_ESM.pdf]

Supplementary Information

Parvalbumin basket cell myelination accumulates axonal mitochondria to internodes

Koen Kole[1], Bas J. B. Voesenek[1], Maria E. Brinia[1,2] & Maarten H. P. Kole[1,3]

[1] Axonal Signaling Group, Netherlands Institute for Neuroscience, Royal Netherlands Academy of Arts and Sciences, Meibergdreef 47, 1105 BA, Amsterdam, the Netherlands

[2] Medical School, National Kapodistrian University of Athens, Athens 11527, Greece

[3] Cell Biology, Neurobiology and Biophysics, Department of Biology, Faculty of Science, Utrecht University, Padualaan 8, 3584 CH, Utrecht, the Netherlands

Supplementary Figures 1-8

Supplementary Tables 1, 2

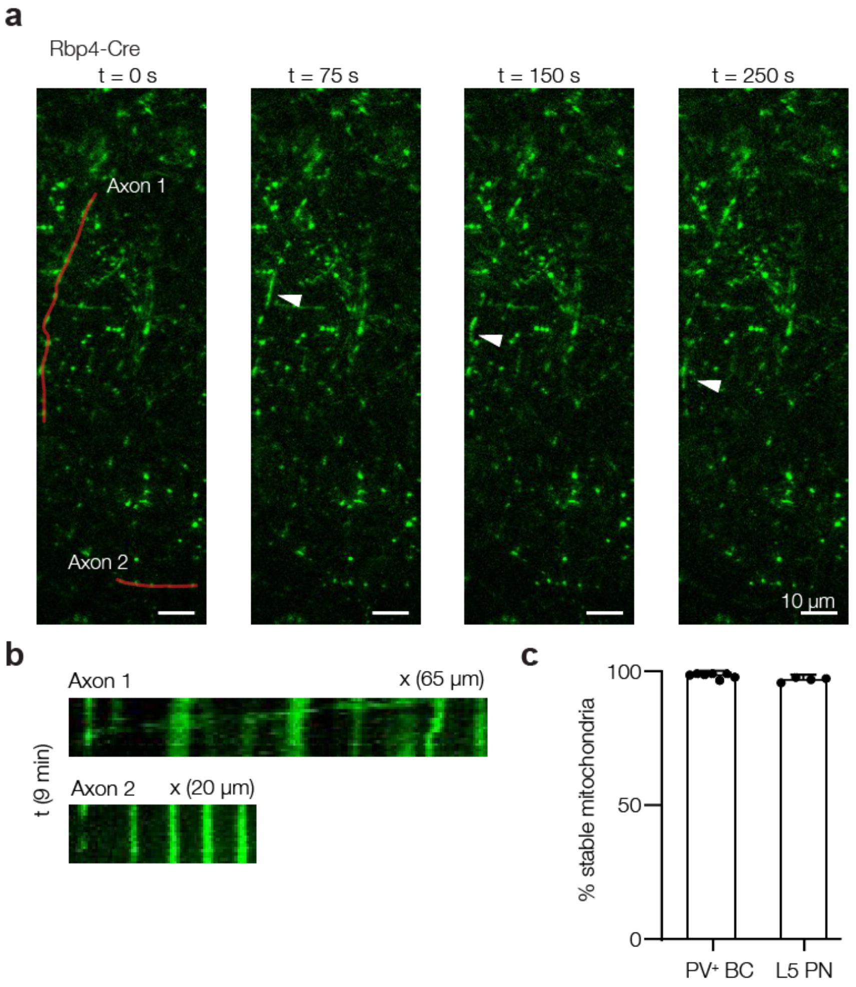

Supplementary Fig. 1 - Mitochondrial motility in acute brain slices a Example frames showing motile mitochondria (mt-GFP, green) in one L5 PN axon, but all other mitochondria are stationary. Red lines indicate paths used for kymographs in b. Similar results were obtained in 7 mice. **b** Example kymographs showing mitochondrial motility (1) and stability (2). **c** Quantification of stationary mitochondria in PV^+ interneurons (PV^+ basket cells (BC); PV-Cre; Ai14 mice, n = 4) or L5 PNs (Rbp4-Cre mice, n = 3). PV-Cre; Ai14, n = 11/1236; Rbp4-Cre, n = 23/1145 motile mitochondria/total mitochondria. Bars indicate means, error bars indicate SEM. Source data are provided as a Source Data file.

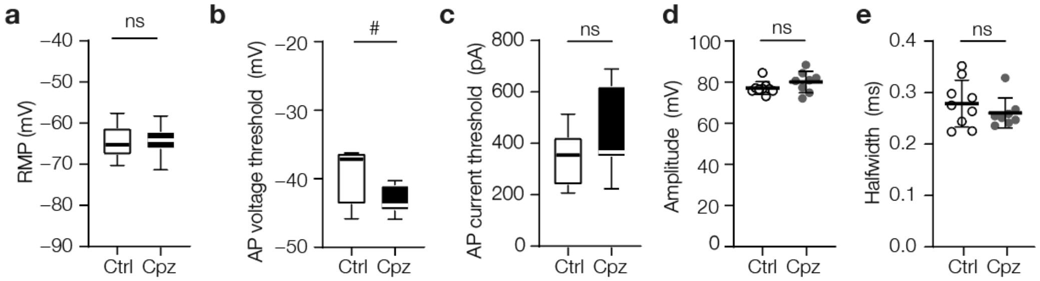

Supplementary Fig. 2 – Action potential parameters and mitochondrial distribution at the AIS upon demyelination. a No change in resting membrane potential (RMP; two-sided unpaired t-test, P = 0.8056; df = 15; control, n = 9 cells from 5 mice; cuprizone, n = 8 cells from 5 mice). **b** Voltage threshold shows a trend towards reduction upon cuprizone-mediated demyelination (two-sided unpaired t-test, P = 0.0590; df = 15; control, n = 9 cells from 5 mice; cuprizone, n = 8 cells from 5 mice); No difference between control and cuprizone-treated groups in **c** current threshold (two-sided unpaired t-test, P = 0.2066), **d** AP amplitude (two-sided unpaired t-test, P = 0.1697; df = 15) or **e** halfwidth (two-sided unpaired t-test, P = 0.3489; df = 15; control, n = 9 cells from 5 mice; cuprizone, n = 8 cells from 5 mice). Horizontal bars indicate the mean, individual data points represent cells, error bars indicate SEM; Box plots represent the 25th to 75th percentiles, whiskers indicate the maximal and minimal values, solid line represents the median. Source data are provided as a Source Data file.

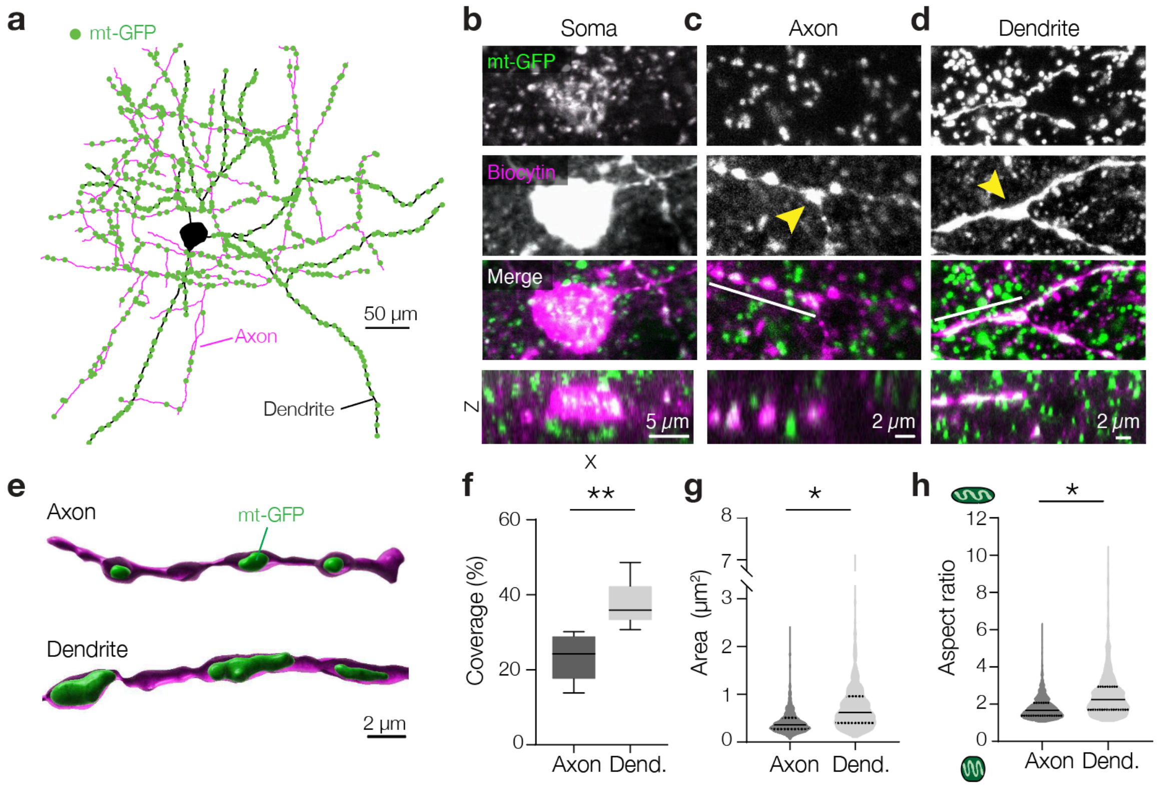

Supplementary Fig. 3 – Axonal and dendritic PV$^+$ interneuron mitochondria are morphologically distinct. a Example of a reconstructed PV$^+$ interneuron and the locations of its mitochondria (green). Magenta, axon; black, dendrite. Mitochondria in soma were not quantified. **b** Examples of somatic, **c** axonal and **d** dendritic mitochondria in PV$^+$ interneurons. Yellow arrowheads indicate branch points, white lines indicate axis used for *XZ* views. Similar results were obtained in 3 mice. **e** 3D surface rendering of example axonal or dendritic mitochondria. **f** Percentage of PV$^+$ neurite length covered by mitochondrial length (two-sided unpaired t-test, $^{**}P = 0.0093$; df = 8; Axon, n = 188 mitochondria; dendrite, n = 482 mitochondria; 5 cells from 3 mice). **g** Area of mitochondria is larger in PV$^+$ dendrites (two-sided nested t-test, $^{*}P = 0.0144$; df = 8; Axon, n = 859 mitochondria; dendrite, n = 1138 mitochondria; 5 cells from 3 mice). **h** Aspect ratio of mitochondria is higher in PV$^+$ dendrites, reflecting a more elongated morphology (two-sided nested t-test, $^{*}P = 0.0318$; df = 8; Axon, n = 859 mitochondria; dendrite, n = 1138 mitochondria; 5 cells from 3 mice). Box plots (f) represent the 25th to 75th percentiles, whiskers represent the maximal and minimal values, solid line represents the median. Solid lines in truncated violin plots (g-h) represent the median, dotted lines represent 25th and 75th quartiles. Source data are provided as a Source Data file.

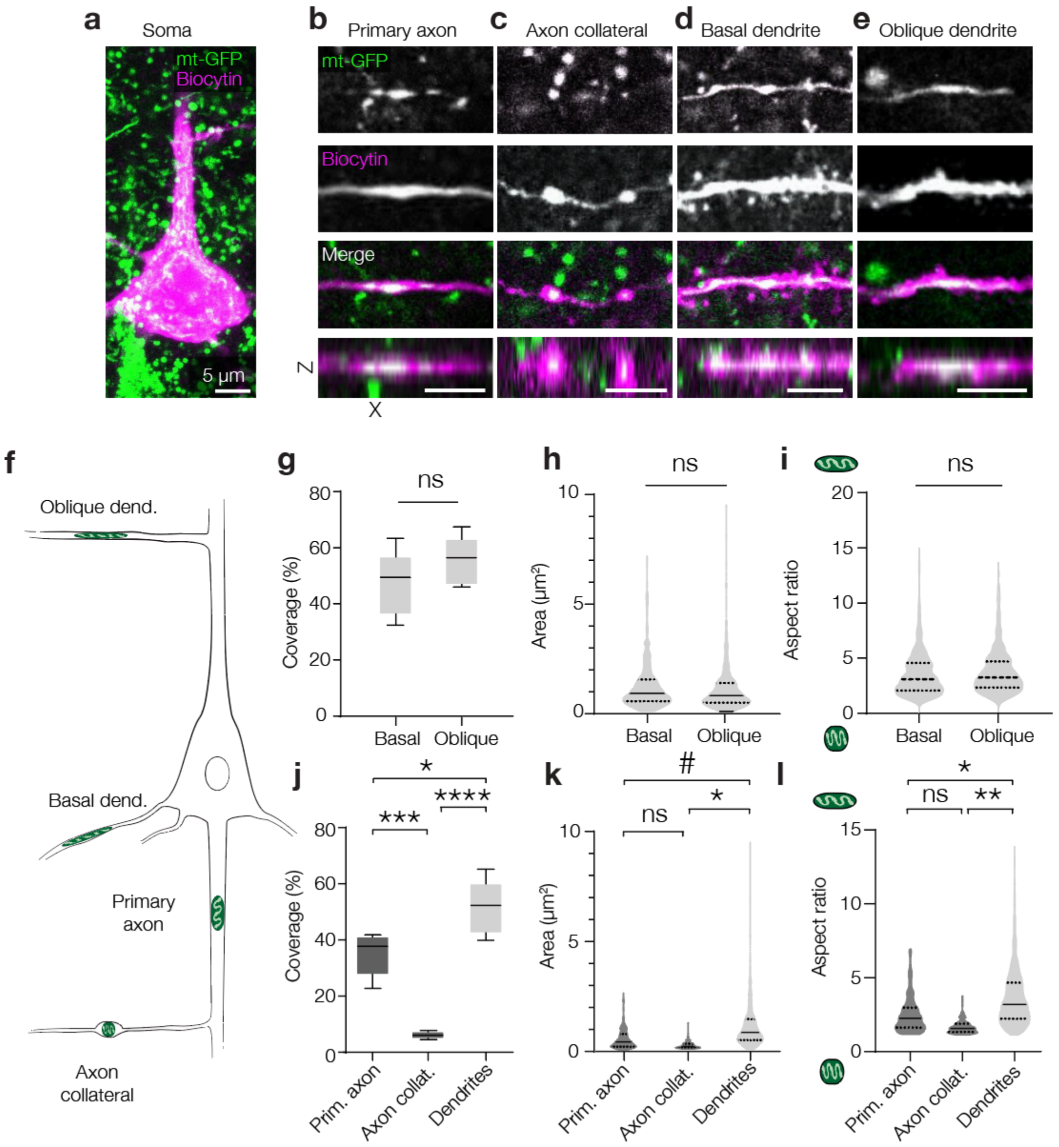

Supplementary Fig. 4 – Axonal mitochondria are distinct from those in dendrites in L5 PNs. Example images of mitoGFP-labeled mitochondria in the **a** soma, **b** primary axon, **c** axon collateral, **d** oblique dendrite branch or **e** basal dendrite of a L5 PN. Somatic mitochondria were not quantified. Similar results were obtained in 3 mice. **f** Schematic of subcellular compartments of the L5 PN that were investigated. **g** Dendritic coverage is not different between basal and oblique dendrites (two-sided unpaired t-test, $P = 0.2538$; df = 8; basal dendrites, $n = 321$; oblique dendrites, $n = 331$ mitochondria from 5 cells of 3 mice each for each). **h** Mitochondria from basal and oblique dendrites have similar surface area (two-sided nested t-test, $P = 0.9554$; df = 8) and **i** aspect ratio (two-sided nested t-test, $P = 0.7162$; df = 8; basal dendrites, $n = 321$; oblique dendrites, $n = 331$ mitochondria from 5 cells of 3 mice each for each). **j** Neurite length covered by mitochondrial length (one-way ANOVA, $P < 0.0001$; Bonferroni's post hoc test, Primary axon vs axon collaterals, ***$P = 0.0001$; primary axon vs dendrites, **$P = 0.0141$; axon collateral vs dendrites, ****$P < 0.0001$; primary axon $n = 188$, axon collateral $n = 125$, dendrites $n = 652$ mitochondria; for each, $n = 5$ cells from 3 mice). **k** Mitochondrial area in L5 PNs is largest in

dendrites and smallest in axon collaterals (nested one-way ANOVA, **P* = 0.0192; Bonferroni's post hoc test, primary axon vs axon collaterals *P* > 0.9999; primary axon vs dendrites, #*P* = 0.0917; axon collaterals vs dendrites, **P* = 0.0241; primary axon *n* = 188, axon collateral *n* = 125, dendrites *n* = 652 mitochondria; for each, *n* = 5 cells from 3 mice). **l** Mitochondria are significantly more round in L5 PN axons compared to dendrites (nested one-way ANOVA, *P* = 0.0031; Bonferroni's post hoc test, primary axon vs axon collaterals, *P* = 0.5094; primary axon vs dendrites, ***P* = 0.0418; axon collaterals vs dendrites, ****P* = 0.0031; primary axon *n* = 188, axon collateral *n* = 125, dendrites *n* = 652 mitochondria; for each, *n* = 5 cells from 3 mice). Box plots represent the 25th to 75th percentiles, whiskers represent the maximal and minimal values, solid line represents the median. In truncated violin plots, solid lines represent the median, dotted lines represent 25th and 75th quartiles. Source data are provided as a Source Data file.

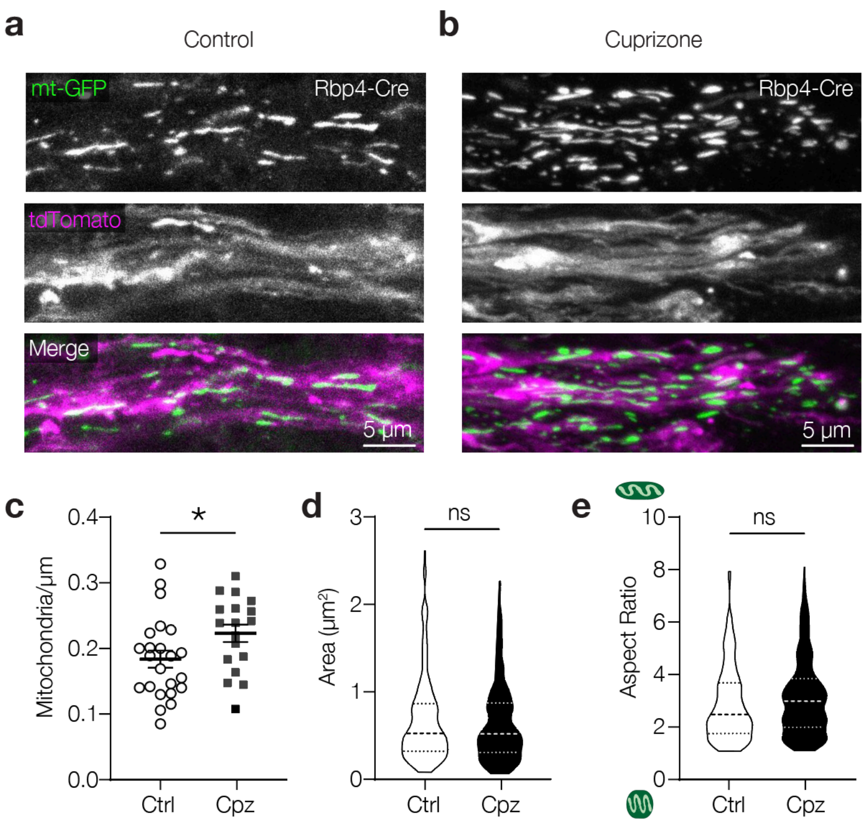

Supplementary Fig. 5 – Increased mitochondrial density in the demyelinated white matter excitatory axons. a, **b** Example confocal images of mitochondria in the white matter of control (a) or cuprizone-treated (b) L5 pyramidal neuron axons from Rbp4-Cre mice. Similar results were obtained in 6 mice. **c** Increased density after cuprizone-mediated demyelination (two-sided unpaired t-test, *$P = 0.0441$; control, $n = 23$ axons from 3 mice; cuprizone, $n = 18$ axons from 3 mice). **d** Mitochondrial size is unchanged after demyelination (two-sided nested t-test, $P = 0.6886$; control, $n = 172$ mitochondria from 23 axons of 3 mice; cuprizone, $n = 162$ mitochondria from 19 axons of 3 mice). **e** Comparable aspect ratio of mitochondria in control or cuprizone-treated white matter axons (two-sided nested t-test, $P = 0.1668$; control, $n = 172$ mitochondria from 23 axons of 3 mice; cuprizone, $n = 162$ mitochondria from 19 axons of 3 mice). Horizontal lines in c the mean, error bars represent SEM, individual data points indicate axons. In truncated violin plots, solid lines represent the median, dotted lines represent 25th and 75th quartiles. Source data are provided as a Source Data file.

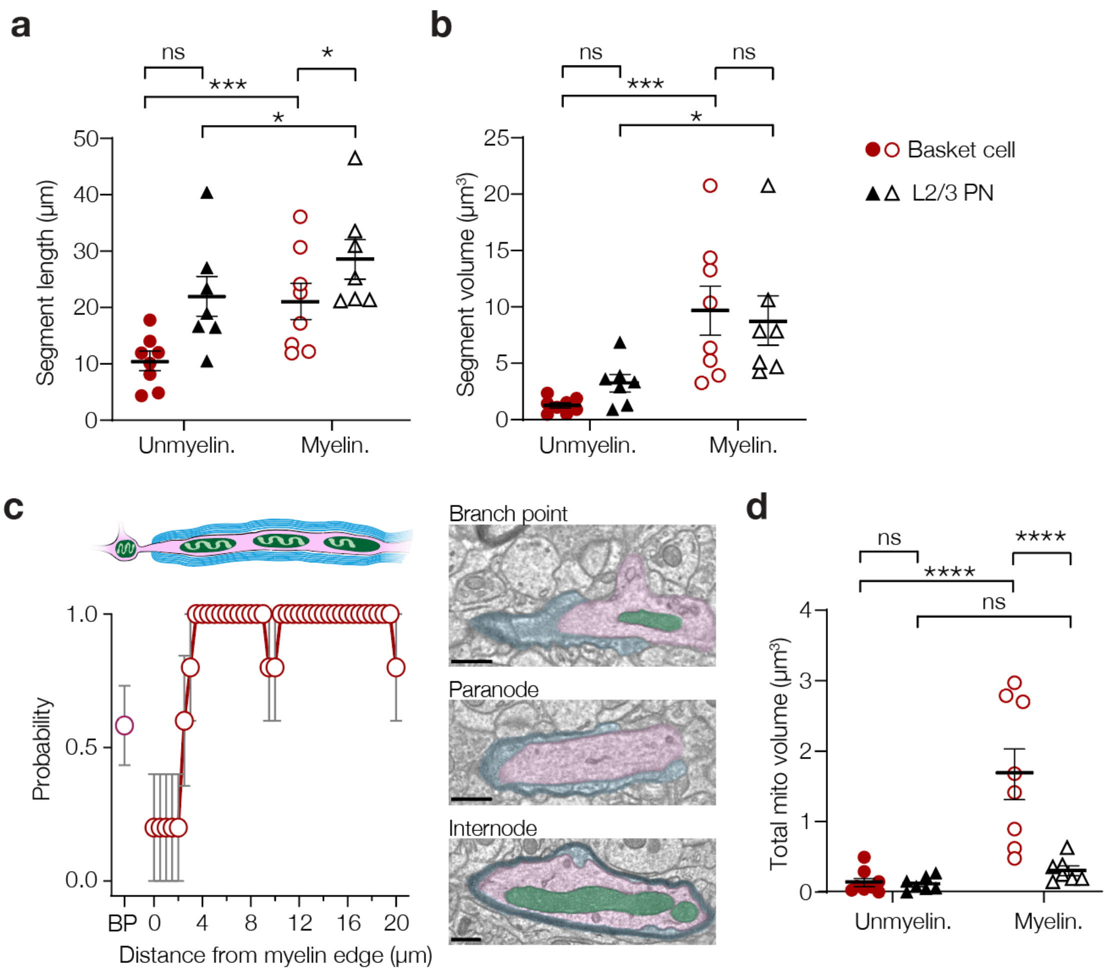

Supplementary Fig. 6 – Myelination-dependent internode properties and mitochondria distribution

a Segment length of (un)myelinated axons in basket cells or L2/3 PNs (two-way ANOVA, myelination x cell type effect, P = 0.2139, $F_{(1,13)}$ = 1.708; myelination effect, P < 0.0001, $F_{(1,13)}$ = 32.78; cell type effect, P = 0.0339, $F_{(1,13)}$ = 5.619; Bonferroni's post hoc test, basket myelinated vs. unmyelinated, ***P = 0.0004; PN myelinated vs. unmyelinated, *P = 0.0195; Myelinated basket vs PN, P = 0.1804; Unmyelinated basket vs PN *P = 0.0253). **b** Segment volume of (un)myelinated axons in basket cells or L2/3 PNs (two-way ANOVA, myelination x cell type effect, P = 0.2765, $F_{(1,13)}$ = 1.209; myelination effect, P = 0.0001, $F_{(1,13)}$ = 28.30; cell type effect, P = 0.7825, $F_{(1,13)}$ = 0.07945; Bonferroni's post hoc test, basket myelinated vs. unmyelinated, ***P = 0.0008; PN myelinated vs. unmyelinated, *P = 0.0265; Myelinated basket vs PN, P > 0.9999; Unmyelinated basket vs PN P = 0.7632). **c** Probability of mitochondria occupancy at branch points (BP; presumed nodes) and along the myelinated internode. *Left:* Mitochondria are often found at branch points (probability 0.58; 12 branch points from 8 cells), show limited occupancy in the first ~2 µm of the internode and are distributed evenly and with high probability along the internode (5 internodes from 4 cells). *Right:* example EM images of a branchpoint

containing a mitochondrion, a paranode devoid of mitochondria and an internode containing a large mitochondrion. Axons, myelin and mitochondria are pseudocoloured (magenta, blue and green, respectively). Scale bar, 500 nm. **d** Total volume of mitochondria in (un)myelinated axons in basket cells or L2/3 PNs (two-way ANOVA, myelination x cell type effect, $P = 0.0027$, $F_{(1,13)} = 13.58$; myelination effect, $P = 0.0004$, $F_{(1,13)} = 22.16$; cell type effect, $P = 0.0052$, $F_{(1,13)} = 11.24$; Bonferroni's post hoc test, basket myelinated vs. unmyelinated, $^{****}P < 0.0001$; PN myelinated vs. unmyelinated, $P = 0.9925$; Myelinated basket vs PN, $^{****}P < 0.0001$; Unmyelinated basket vs PN $P > 0.9999$). Scale bars for EM images in c represent 0.5 µm. Data points in a and b represent axons (basket, $n = 8$ cells; PN, $n = 7$ cells; 1-4 segments per cell for each cell type), data points in d represent total mitochondrial volume per axon (basket, $n = 8$ cells, $n = 68$ mitochondria in myelinated and 18 mitochondria in unmyelinated segments; PN, $n = 7$ cells; $n = 21$ mitochondria in myelinated and 53 in unmyelinated segments). Solid horizontal bars indicate means, error bars indicate SEM. Source data are provided as a Source Data file. Source data are provided as a Source Data file.

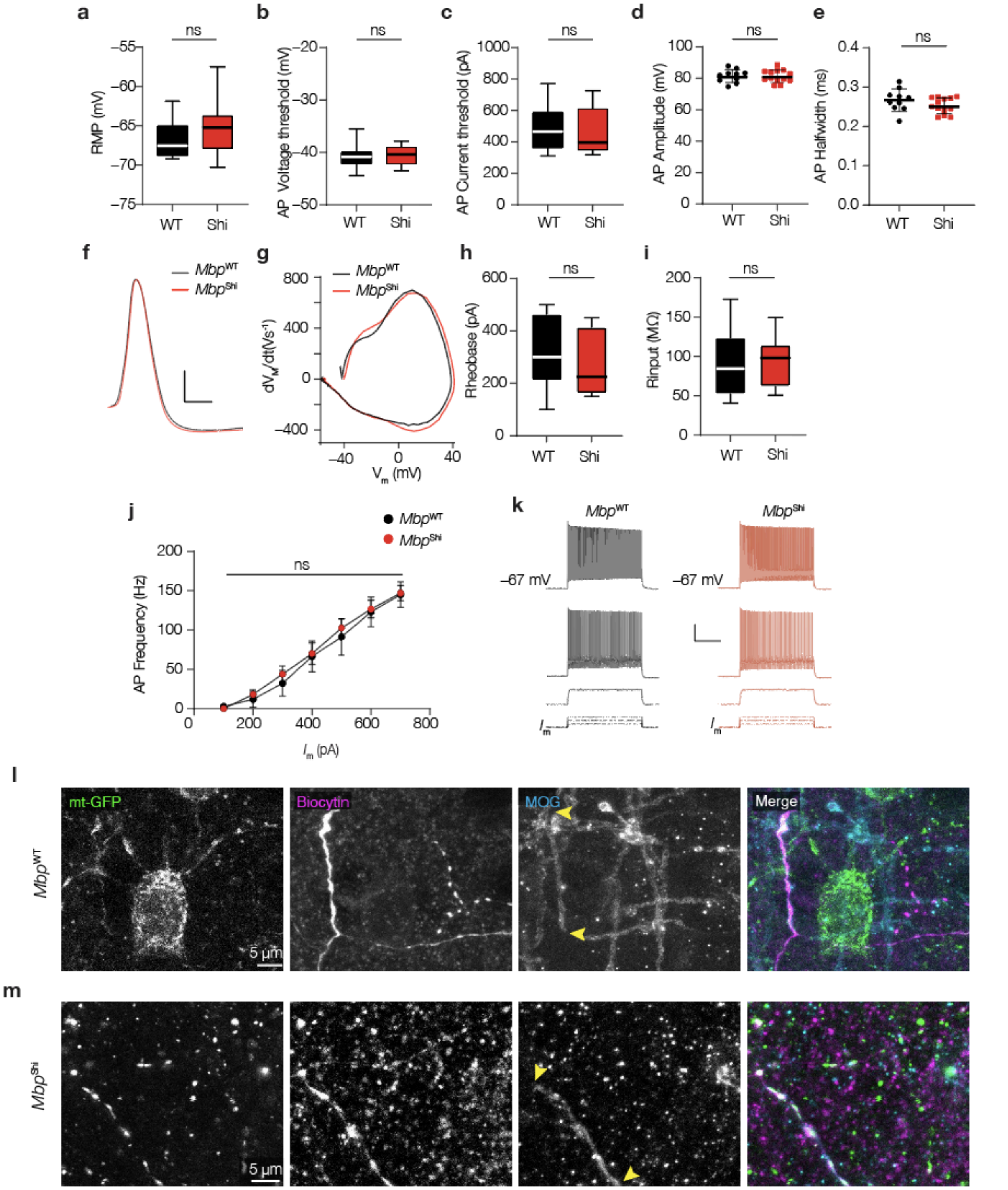

Supplementary Fig. 7 – Electrophysiological properties of PV$^+$ interneurons in Mbp^{WT} or Mbp^{Shi} mice. a Resting membrane potential (two-sided unpaired t-test, P = 0.2597; df = 18; Mbp^{WT}, n = 9 cells from 3 mice; Mbp^{Shi}, n = 13 cells from 3 mice); **b** Action potential membrane threshold (two-sided unpaired t-test, P = 0.8364; df = 21; Mbp^{WT}, n = 10 cells from 3 mice; Mbp^{Shi},

n = 13 cells from 3 mice); **c** Action potential current threshold (two-sided unpaired t-test, *P* = 0.6566; df = 21; Mbp^{WT}, *n* = 10 cells from 3 mice; Mbp^{Shi}, *n* = 13 cells from 3 mice); **d** Action potential amplitude (two-sided unpaired t-test, *P* = 0.9358; df = 21; Mbp^{WT}, *n* = 10 cells from 3 mice; Mbp^{Shi}, *n* = 13 cells from 3 mice); **e** Action potential halfwidth (-two-sided unpaired t-test, *P* = 0.1744; df = 21; Mbp^{WT}, *n* = 10 cells from 3 mice; Mbp^{Shi}, *n* = 13 cells from 3 mice); **f** Example single action potential from either genotype, scalebar indicates 10 mV and 0.25 ms; **g** Phase-plane plots of the example traces in h; **h** Rheobase (two-sided unpaired t-test, *P* = 0.4395; df = 18; Mbp^{WT}, *n* = 8 cells from 3 mice; Mbp^{Shi}, *n* = 12 cells from 3 mice); **i** Input resistance (two-sided unpaired t-test, *P* = 0.8375; df = 18; Mbp^{WT}, *n* = 8 cells from 3 mice; Mbp^{Shi}, *n* = 12 cells from 3 mice); **j** Comparable action potential firing frequency in Mbp^{WT} or Mbp^{Shi} mice (two-way ANOVA, *P* = 0.9967, $F_{(13, 221)}$ = 0.2488; genotype effect *P* = 0.7752, $F_{(1, 17)}$ = 0.08422; Mbp^{WT}, *n* = 7 cells from 3 mice; Mbp^{Shi}, *n* = 12 cells from 3 mice); **k** Example traces in response to 200, 400 or 600 pA somatic current injection, scale bar indicates 25 mV and 250 ms.). **l, m** Confocal images corresponding to the 3D renders in Fig. 6. Yellow arrowheads indicate internodes. Similar results were obtained in 6 mice. Individual data points in a-k represent cells, error bars indicate SEM; Box plots represent the 25th to 75th percentiles, whiskers indicate the maximal and minimal values, solid line represents the median. Source data are provided as a Source Data file.

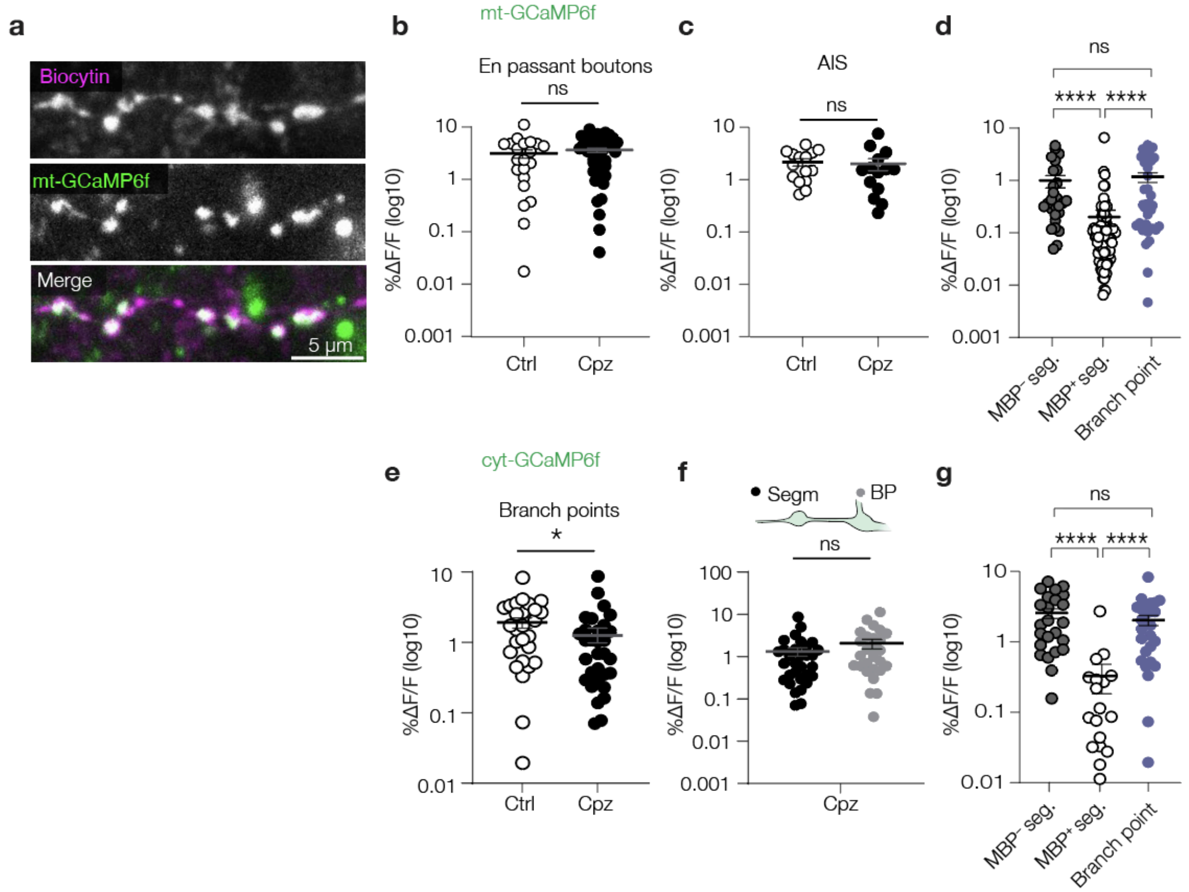

Supplementary Fig. 8 – mt-Ca^{2+} and cyt-Ca^{2+} responses in PV^{+} axons

a Example confocal image of PV^{+} *en passant* boutons. Similar results were obtained in 4 mice. **b** Demyelination does not alter mt-Ca^{2+} buffering in *en passant* boutons (two-sided Mann-Whitney test, P = 0.2847; control, n = 21 mitochondria of 4 cells from 2 mice; cuprizone, n = 47 mitochondria of 8 cells from 4 mice). **c** mt-Ca^{2+} responses at the AIS are not changed after demyelination (two-sided Mann-Whitney test, P = 0.1694; control, n = 13 mitochondria of 5 AISs from 3 mice; cuprizone, n = 20 mitochondria of 6 AISs from 4 mice). **d** In control PV^{+} axons, amplitude of mt-Ca^{2+} responses at MBP^{-} segments are not different from those at branch points (Kruskal-Wallis test, ****P < 0.0001; Dunn's post hoc test, MBP^{+} vs MBP^{-} ****P < 0.0001, MBP^{-} vs. branch points P > 0.9999; MBP^{+} vs branch points P < 0.0001; MBP^{+} segments, n = 101 mitochondria of 12 cells from 7 mice, same data as in Fig. 7d; MBP^{-} segments, n = 27 mitochondria of 9 cells from 5 mice, same data as in Fig. 7d; branch points, n = 38 mitochondria of 11 cells from 7 mice, same data as in Fig. 8f); **e** Smaller cyt-Ca^{2+} transients in branch points after demyelination (two-sided Mann-Whitney test, *P = 0.0307; control, n = 27 branch points of 7 cells from 3 mice; cuprizone, n = 31 branch points of 6 cells from 3 mice); **f** Cytosolic Ca^{2+} responses are comparable in segments and branch points upon demyelination (two-sided Mann-Whitney test, P = 0.2476; n = 28 segments of 6 cells from 3 mice, n = 31 branch points of 6 cells from 3 mice, same data as in e); **g** In control PV^{+} axons, amplitude of cytosolic Ca^{2+} responses at MBP^{-} segments are not different from those at branch points (Kruskal-Wallis test, ****P < 0.0001; Dunn's post hoc test, MBP^{+} vs MBP^{-} ****P < 0.0001, MBP^{-} vs. branch points P > 0.9999; MBP^{+} vs branch points P < 0.0001; MBP^{+}, n = 18 segments of 6 cells from 3 mice; MBP^{-}, n = 24

segments of 8 cells from 3 mice, same data as in Fig. 7f; branch points, control, *n* = 27 branch points of 7 cells from 3 mice, same data as in e). Individual data points in b-d indicate mitochondria, individual data points in e-g indicate segments or branch points as indicated. Horizontal bars indicate means, error bars indicate SEM. Source data are provided as a Source Data file.

Supplementary Tables

Supplementary Table 1

Antibody	Host	Dilution	Manufacturer	Cat. number	RRID
Anti-Green fluorescent protein	Chicken	1:500	Abcam	ab13970	AB_300798
Anti-Red fluorescent protein	Mouse	1:500	Abcam	ab65856 (discontinued)	AB_1141717
Anti-βIV Spectrin	Rabbit	1:1000	Engelhardt and Kole lab	N/A	N/A
Anti-Myelin Basic Protein	Mouse	1:250	Covance	SMI-99P	AB_10120129
Anti-Myelin Oligodendrocyte Glycoprotein	Mouse	1:250	Millipore	MAB5680	RRID:AB_1587278
Anti-Mouse-Alexa-405	Goat	1:500	ThermoFisher	A31553	AB_221604
Anti-Chicken-Alexa-488	Goat	1:500	ThermoFisher	A11039	AB_142924
Anti-Rabbit-Alexa-633	Goat	1:500	ThermoFisher	A21070	AB_2535731
Anti-Mouse-Alexa-647	Goat	1:500	ThermoFisher	A21235	AB_2535804
Streptavidin-Alexa-594	N/A	1:500	ThermoFisher	S11227	N/A
Streptavidin-Alexa-633	N/A	1:500	ThermoFisher	S32364	AB_2313500

Overview of antibodies and dilutions used in this study.

Supplementary Table 2

Cell name	Neuroglancer ID	Cell type	Location of interest
PV1_1	864691135307240262	Basket cell	174508, 124118, 21157
PV1_2	864691135307240262	Basket cell	180056, 128599, 21514
PV2_1	864691135367308281	Basket cell	178363, 187957, 20579
PV3_1	864691135386593409	Basket cell	181574, 159259, 20999
**PV3_2**	**864691135386593409**	**Basket cell**	**191862, 145103, 20142**
PV4_1	864691135544579880	Basket cell	171765, 173397, 20706
PV5_1	864691135428608048	Basket cell	181752, 143942, 22968
PV6_1	864691135590150923	Basket cell	170564, 132985, 19749
PV7_1	864691135697462549	Basket cell	173603, 134391, 22067
PV8_1	864691136952074207	Basket cell	186366, 118464, 20183
PN_1	864691135855890478	L2/3 PN	144874, 149452, 23959
PN_2	864691135975539779	L2/3 PN	337852, 147425, 19387
PN_3	864691135564739159	L2/3 PN	334986, 157943, 20515
PN_4	864691136031786427	L2/3 PN	123284, 152471, 23565
PN_5	864691136618414989	L2/3 PN	222968, 137995, 23424
PN_6	864691135012580342	L2/3 PN	238983, 137249, 23366
**PN_7**	**864691135099882016**	**L2/3 PN**	**241318, 147339, 23370**

Overview of the cells used in the 3D EM analysis. Bold marked cells were used as examples in **Figure 5**.
