# Supplementary material for: Parvalbumin basket cell myelination accumulates axonal mitochondria to internodes
Source: Nat Commun. 2022 Dec 9;13:7598. doi: 10.1038/s41467-022-35350-x (PMC9734141; doi:10.1038/s41467-022-35350-x)
Supplement: Supplementary file 3 — Description of Additional Supplementary Files [file 41467_2022_35350_MOESM3_ESM.pdf]

Description of Additional Supplementary Files

Supplementary Movie 1: Heatmap of mitochondrial Ca2+ responses in a PV+ axons upon 100 APs. A significant response can be seen in the branch point but not under the myelin sheath. Video corresponds to Fig. 7b.

Supplementary Movie 2: Heatmap of cytosolic Ca2+ responses in a PV+ axons upon 100 APs. In the AIS and the first branch point, significant responses are seen but not under the myelinated internode. Video corresponds to Fig. 7e.
