# Supplementary material for: Parvalbumin basket cell myelination accumulates axonal mitochondria to internodes
Source: Nat Commun. 2022 Dec 9;13:7598. doi: 10.1038/s41467-022-35350-x (PMC9734141; doi:10.1038/s41467-022-35350-x)
Supplement: Supplementary file 6 — Reporting Summary [file 41467_2022_35350_MOESM6_ESM.pdf]

nature portfolio

Corresponding author(s): Maarten Kole
Koen Kole

Last updated by author(s): Nov 14, 2022

Reporting Summary

Nature Portfolio wishes to improve the reproducibility of the work that we publish. This form provides structure for consistency and transparency in reporting. For further information on Nature Portfolio policies, see our Editorial Policies and the Editorial Policy Checklist.

Statistics

For all statistical analyses, confirm that the following items are present in the figure legend, table legend, main text, or Methods section.

n/a	Confirmed	
☐	☒	The exact sample size (*n*) for each experimental group/condition, given as a discrete number and unit of measurement
☐	☒	A statement on whether measurements were taken from distinct samples or whether the same sample was measured repeatedly
☐	☒	The statistical test(s) used AND whether they are one- or two-sided<br>*Only common tests should be described solely by name; describe more complex techniques in the Methods section.*
☐	☒	A description of all covariates tested
☐	☒	A description of any assumptions or corrections, such as tests of normality and adjustment for multiple comparisons
☐	☒	A full description of the statistical parameters including central tendency (e.g. means) or other basic estimates (e.g. regression coefficient) AND variation (e.g. standard deviation) or associated estimates of uncertainty (e.g. confidence intervals)
☐	☒	For null hypothesis testing, the test statistic (e.g. *F*, *t*, *r*) with confidence intervals, effect sizes, degrees of freedom and *P* value noted<br>*Give P values as exact values whenever suitable.*
☒	☐	For Bayesian analysis, information on the choice of priors and Markov chain Monte Carlo settings
☒	☐	For hierarchical and complex designs, identification of the appropriate level for tests and full reporting of outcomes
☒	☐	Estimates of effect sizes (e.g. Cohen's *d*, Pearson's *r*), indicating how they were calculated

Our web collection on statistics for biologists contains articles on many of the points above.

Software and code

Policy information about availability of computer code

Data collection	Electrophysiological recordings were performed using AxoGraph X software, version 1.5.4<br>Leica Application Suite AF software version 3.5.7.23225 was used to control confocal imaging<br>Femtonics MES software version 6.3.7902 controlled two-photon imaging<br>3D EM data and cytosol segmentations were obtained from https://www.microns-explorer.org/ ("cortical mm^3" dataset)
Data analysis	Electrophysiological recordings were analyzed using AxoGraph X software, version 1.7.4<br>Two-photon calcium imaging data was analyzed using custom Matlab code; motility was analyzed using Imaris version 9.7.2<br>Cellular reconstructions (confocal images) were performed using MBF Neurolucida version 2019.2.1 or 2020.1.3<br>Mitochondria in 3D EM data were manually segmented using VAST tools version 1.4.1<br>Statistical analysis were done in Graphpad Prism version 8.4.3<br>Extraction of partial images for figures was done using FIJI (64 bit version downloaded 09/07/2021; ImageJ version 1.53q)<br>Calcium data was analyzed using custom written Matlab scripts available at https://github.com/Kolelab. Example data and pseudocode are also available there

For manuscripts utilizing custom algorithms or software that are central to the research but not yet described in published literature, software must be made available to editors and reviewers. We strongly encourage code deposition in a community repository (e.g. GitHub). See the Nature Portfolio guidelines for submitting code & software for further information.

Data

Policy information about availability of data

All manuscripts must include a data availability statement. This statement should provide the following information, where applicable:

- Accession codes, unique identifiers, or web links for publicly available datasets
- A description of any restrictions on data availability
- For clinical datasets or third party data, please ensure that the statement adheres to our policy

Data will be deposited on the servers of the Netherlands Institute for Neuroscience and will be available upon request.
3D EM data is available at www.microns-explorer.org

Field-specific reporting

Please select the one below that is the best fit for your research. If you are not sure, read the appropriate sections before making your selection.

[x] Life sciences [ ] Behavioural & social sciences [ ] Ecological, evolutionary & environmental sciences

For a reference copy of the document with all sections, see nature.com/documents/nr-reporting-summary-flat.pdf

Life sciences study design

All studies must disclose on these points even when the disclosure is negative.

Sample size	We used a power analysis to calculate the number of recordings of individual cells and animals required to reach a power of 0.8 and an alpha of 0.05. Nested t-tests or nested one-way ANOVAs were used when large numbers of datapoints were involved (i.e. mitochondrial contours) to avoid overpowering of non-nested statistical tests. Cells were nested inside their respective treatment groups.
Data exclusions	Electrophysiological data was excluded if cells had an unstable resting membrane potential between sweeps.<br>Calcium data was excluded when the number of action potentials deviated by more than 15% (<85 or >115 APs) or if the subsequent immunostaining was not reliable.
Replication	Data were collected from multiple cells from multiple animals to ensure reproducibility. Experiments were replicated with a minimum of 4 cells from a minimum of 3 mice. For exact n numbers see figure legends. Reconstructions and recordings from multiple subcellular compartments of the same cell were obtained wherever possible. The 3D EM reconstructions are an exception as these data was obtained from one mouse.
Randomization	Mice were randomly allocated to either the control or cuprizone treatment. The researcher who determined which animals went into which group did not see the animals beforehand and allocated them solely on the basis of their sex, age, weight and genotype. In the case of Shiverer mice, genotype (MBP-WT/MBP-Shi) was leading in determining group allocation but also here the researcher did not see the animals prior to allocation. For comparisons of subcellular compartments (e.g. myelinated vs unmyelinated axon, axon vs dendrite, segment vs branch point), randomization is not possible as the group data belong to the same cell. Selection of cells for patching prior to e.g. cellular reconstruction and calcium imaging was done randomly as much as possible (i.e. virally transduced cells with a healthy appearance were targeted but otherwise no selection was made).
Blinding	Blinding was not performed in this study as the difference between control and cuprizone-treated brain tissue (or brain tissue from Shiverer mice) can be readily observed during the performance of the experiments (i.e. due to the absence of myelin). In control cells, calcium imaging was done blind to the myelination state of axonal segments, which was revealed only later by immunohistochemistry. To determine the density of mitochondria in MBP+/MOG+ and MBP-/MOG- segments, during tracing of the axon and mitochondria the MBP or MOG signal was turned off and vice versa. This was not possible in the 3D EM dataset, where the presence of myelin is readily observed in the image data that is required to perform the segmentation, so blinding was not possible in this case.

Reporting for specific materials, systems and methods

We require information from authors about some types of materials, experimental systems and methods used in many studies. Here, indicate whether each material, system or method listed is relevant to your study. If you are not sure if a list item applies to your research, read the appropriate section before selecting a response.

Materials & experimental systems

n/a	Involved in the study
[ ]	[x] Antibodies
[ ]	[x] Eukaryotic cell lines
[x]	[ ] Palaeontology and archaeology
[ ]	[x] Animals and other organisms
[x]	[ ] Human research participants
[x]	[ ] Clinical data
[x]	[ ] Dual use research of concern

Methods

n/a	Involved in the study
[x]	[ ] ChIP-seq
[x]	[ ] Flow cytometry
[x]	[ ] MRI-based neuroimaging

Antibodies

Antibodies used

All information about the antibodies including supplier, catalog number, used concentration, lot number and RRID are detailed in the supplemental materials, Table S1

Validation

Anti-Green fluorescent protein: validated by Abcam via immunofluorescence and western blot (ab13970)
Anti-Red fluorescent protein: validated by Abcam (ab65856)
Anti-βIV Spectrin: validated using knockout, immunofluorescence and western blot (see Jamann et al., Nat. Comm. 2021)
Anti-Myelin Basic Protein: validated by Covance via immunofluorescence and western blot (SMI-99P)
Anti-Myelin Oligodendrocyte Glycoprotein: validated by Millipore via western blot (MAB5680)

Eukaryotic cell lines

Policy information about cell lines

Cell line source(s)

HEK-293T: ATCC

Authentication

HEK-293T: STR profiling

Mycoplasma contamination

Cell lines were not tested for mycoplasma contamination

Commonly misidentified lines
(See ICLAC register)

HEK cells are listed in the ICLAC database. They are however needed for the efficient production of viral vectors, and are commonly used for this purpose.

Animals and other organisms

Policy information about studies involving animals; ARRIVE guidelines recommended for reporting animal research

Laboratory animals

Male and female PV-Cre × Ai14, Rbp4-Cre or Rbp4-Cre × ChETA mice were used in this study. Age ranged from 13 to 14 weeks at sacrifice.

PV-Cre × Ai14 mice were obtained by crossing B6;129S6-Gt(ROSA)26Sortm14(CAG-tdTomato)Hze/J (RRID:IMSR_JAX:007908) with B6;129P2-Pvalbtm1(cre)Arbr/J (RRID:IMSR_JAX:008069)

Rbp4-Cre × ChETA mice were obtained by crossing B6.FVB(Cg)-Tg(Rbp4-cre)KL100Gsat/Mmucd (RRID:MMRRC_037128-UCD) with B6;129-Gt(ROSA)26Sortm1(CAG-COP4*E123T*H134R,-tdTomato)Gfng/J (RRID:IMSR_JAX:017455)

PV-Cre × Ai14 × Shiverer mice were obtained by crossing PV-Cre × Ai14 mice (see above) with C3Fe.SWV-MbpShi/J (RRID:IMSR_JAX:001428)

Wild animals

Not applicable

Field-collected samples

Not applicable

Ethics oversight

All procedures were performed after evaluation by the KNAW Animal Ethics Committee (DEC) and Central Authority for Scientific Procedures on Animals (CCD, license AVD8010020172426). The specific experimental designs were evaluated and monitored by the Animal Welfare Body (IvD, protocols NIN19.21.01, NIN19.21.09 and NIN19.21.12).

Note that full information on the approval of the study protocol must also be provided in the manuscript.
